# Identification of seven‐gene marker to predict the survival of patients with lung adenocarcinoma using integrated multi‐omics data analysis

**DOI:** 10.1002/jcla.24190

**Published:** 2021-12-23

**Authors:** Surong Zhang, Xueni Zeng, Shaona Lin, Minchao Liang, Huaxing Huang

**Affiliations:** ^1^ Department of Infectious Diseases The Second Affiliated Hospital of Guangzhou Medical University Guangzhou City China; ^2^ Department of Pulmonary and Critical Care Medicine The Second Affiliated Hospital of Guangzhou Medical University Guangzhou City China; ^3^ Department of Medicine Shenzhen Haplox Biotechnology Co., Ltd Shenzhen City China

**Keywords:** bioinformatics, CNV, lung adenocarcinoma, prognostic markers, TCGA

## Abstract

**Background:**

The mechanism of cancer occurrence and development could be understood with multi‐omics data analysis. Discovering genetic markers is highly necessary for predicting clinical outcome of lung adenocarcinoma (LUAD).

**Methods:**

Clinical follow‐up information, copy number variation (CNV) data, single nucleotide polymorphism (SNP), and RNA‐Seq were acquired from The Cancer Genome Atlas (TCGA). To obtain robust biomarkers, prognostic‐related genes, genes with SNP variation, and copy number differential genes in the training set were selected and further subjected to feature selection using random forests. Finally, a gene‐based prediction model for LUAD was validated in validation datasets.

**Results:**

The study filtered 2071 prognostic‐related genes and 230 genomic variants, 1878 copy deletions, and 438 significant mutations. 218 candidate genes were screened through integrating genomic variation genes and prognosis‐related genes. 7 characteristic genes (RHOV, CSMD3, FBN2, MAGEL2, SMIM4, BCKDHB, and GANC) were identified by random forest feature selection, and many genes were found to be tumor progression‐related. A 7‐gene signature constructed by Cox regression analysis was an independent prognostic factor for LUAD patients, and at the same time a risk factor in the test set, external validation set, and training set. Noticeably, the 5‐year AUC of survival in the validation set and training set was all ˃ 0.67. Similar results were obtained from multi‐omics validation datasets.

**Conclusions:**

The study builds a novel 7‐gene signature as a prognostic marker for the survival prediction of patients with LUAD. The current findings provided a set of new prognostic and diagnostic biomarkers and therapeutic targets.

## Graphical abstract

The study determined 2071 prognostic‐related genes and 230 genomic variants, 1878 copy deletions, and 438 significant mutations. 218 candidate genes in total were screened through integrating genomic variation genes and prognosis‐related genes. 7 characteristic genes (RHOV, CSMD3, FBN2, MAGEL2, SMIM4, BCKDHB, and GANC) were identified by random forest feature selection, and many genes are tumor progression‐related. A 7‐gene signature constructed by Cox regression analysis was an independent prognostic factor for LUAD patients, and a risk factor stratified in the test set, and external validation set, and training set. Noticeably, the 5‐year AUC of survival in the validation set and training set were all ˃ 0.67. The study builds a novel 7‐gene signature as a prognostic marker for the survival prediction of patients with LUAD. The current findings provided a set of new prognostic and diagnostic biomarker and therapeutic target.

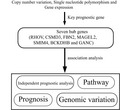

## 1 INTRODUCTION

Lung cancer is a major cause of cancer‐associated death worldwide,^1^ and risk factors cover family history, age, smoking, and air pollution.^2^ Two histological types of lung cancer are small‐cell lung cancer (SCLC) and non‐small‐cell lung cancer (NSCLC).^3^, ^4^ Lung adenocarcinoma (LUAD) is the main type of NSCLC,^5^ and its incidence has been increasing steadily in the past few decades. Tumor, lymph node, and metastasis (TNM) phase is currently the most widely used system to assess the clinical outcome of cases with LUAD.^6^ However, the prognosis of LUAD patients with the same pathologic stage varies greatly.^7^, ^8^, ^9^, ^10^ Currently, an effective system for predicting the outcome of LUAD is needed to help clinicians evaluate treatment outcomes and promote personalized therapy.

A number of studies were conducted for finding biomarkers predictive of long‐term LUAD survival. Biological markers are mainly categorized into several classes, firstly, single molecule as a separated prognosis‐related index, covering squamous cell antigen (SCC), CA125, or other new markers; secondly, many genetic markers as prognostic genes identified using gene expression profiles with high‐throughput screening. There are several systematic biological methods for identifying gene biomarkers associated with LUAD prognosis and constructing genetic features. For instance, Wang et al established a 4‐gene signature based on gene expression profiling using least absolute shrinkage and selection operator (LASSO) selection operator Cox regression and weighted gene correlation network analysis^11^; Liu et al. used a GLYCOLYSIS‐related genes to identify a 4‐gene signature applying Cox proportional hazard regression^12^; Chen et al. built a 11‐gene signature by meta‐analysis of gene expressions^13^; Li et al. developed a 3‐gene signature using network biology method analysis.^14^ Noticeably, these above gene signatures have been independently verified in external datasets, but have not been clinically applied. Thus, it is crucial to analyze the biological function more comprehensively to identify genes associated with LUAD prognosis.

To effectively build a reliable LUAD prognostic process‐associated gene signature, this study developed a scientific pipeline for identifying LUAD‐associated gene markers. Single nucleotide mutations, gene expression profiles, and copy number variation data for LUAD patients were acquired from GEO and TCGA databases. A 7‐gene signature was established by integrating transcriptomics and genomics data to screen prognostic markers. The ability of the signature to predict LUAD survival was validated in external validation and internal test sets. The results validated that the 7‐gene signal participated in vital biology courses and pathways in LUAD. Similarly, GSEA analysis also showed consistent outcomes. The current data indicated that the 7‐gene signature could efficiently estimate cancer prognostic of LUAD patients, providing a better understanding of the molecular mechanism of LUAD prognosis.

## 2 MATERIALS AND METHODS

### 2.1 Data collection and processing

**FIGURE 1:**
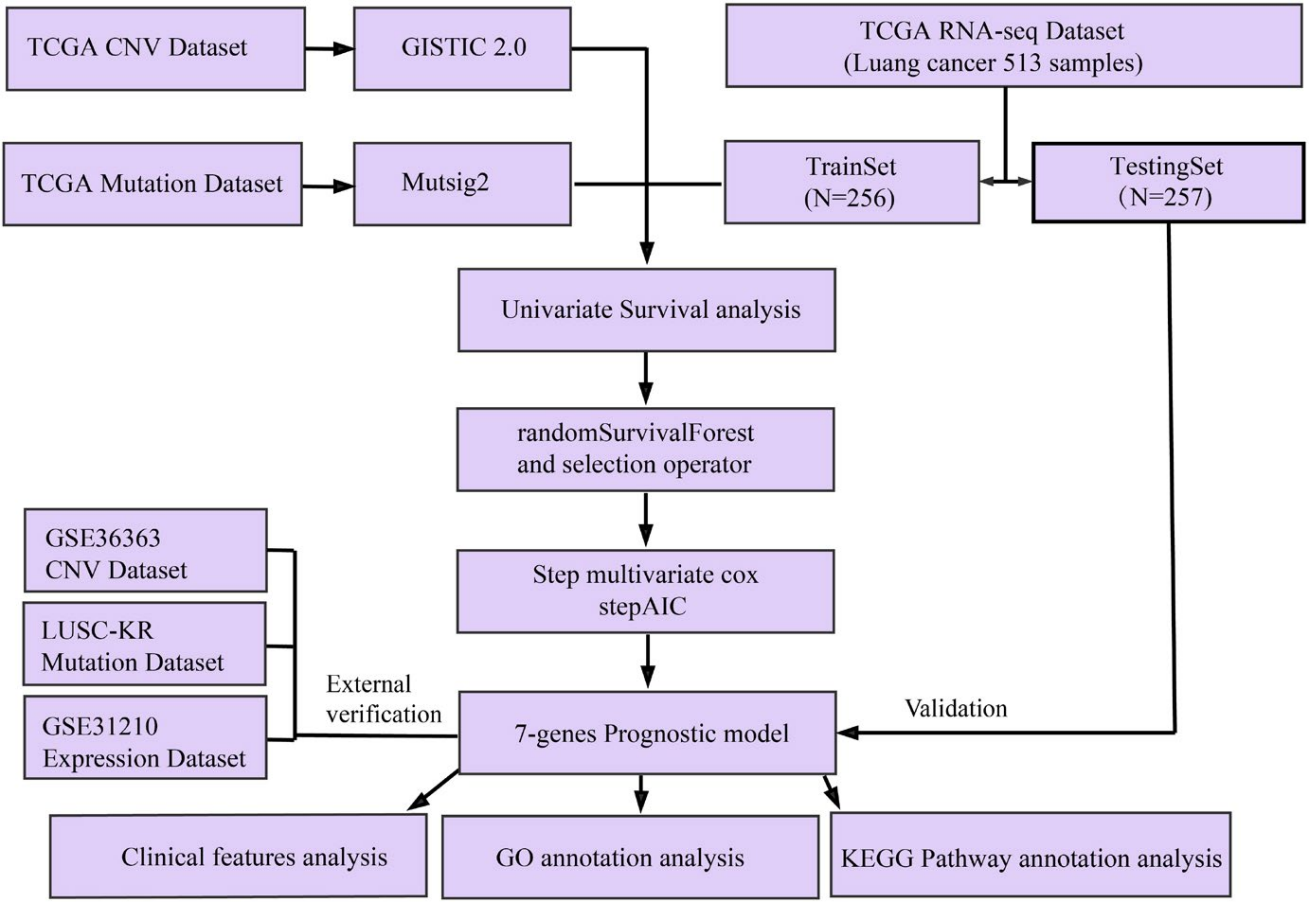
Work flow chart

A total of 516 samples containing SNP 6.0 chip copy number variation data, 576 FPKM samples containing RNA‐Seq data, 738 samples containing clinical follow‐up information were obtained from the UCSC Cancer Browser (https://xenabrowser.net/datapages/). 543 samples of mutation annotation information (MAF) were downloaded from GDC client. On May 25, 2019, the GSE31210^15^ dataset containing a total of 246 samples with standardized expression characteristics and clinic‐related data was downloaded from GEO. In the GSE31210, 226 samples with clinical follow‐up information were acquired. From the TCGA RNA‐Seq data, follow‐up information of 513 LUAD samples was filtered and randomly divided into 2 groups, one group as a training set (*N* = 256) and another as a test set (*N* = 257). All these samples were surgical cases collected before the first treatment. Samples with follow‐up information in the GSE31210 dataset served as external validation sets. Copy number variation (CNV) dataset, GSE36363 (https://www.ncbi.nlm.nih.gov/geo/query/acc.cgi?acc=GSE36363), mutation dataset, and LUSC‐KR (https://dcc.icgc.org/projects/LUSC‐KR) were used as multi‐omics validation datasets. For sample information of each group, see Table 1. The flow chart of this research is shown in Figure 1.

**TABLE 1:** Clinical information statistics of three sets of datasets

Characteristic		TCGA training datasets (*n* = 256)	TCGA test datasets (*n* = 257)	GSE31210 (*n* = 226)
Age(years)	<=50	29	19	27
	>50	227	236	199
Survival Status	Living	161	167	191
	Dead	95	90	35
Gender	Female	135	141	121
	Male	121	116	105
Pathologic_T	T 1	82	89	
	T 2	141	134	
	T 3	24	22	
	T 4	7	11	
Pathologic_N	N 0	166	170	
	N 1	53	41	
	N 2	31	38	
	N 3	1	1	
Pathologic_M	M 0	174	169	
	M 1/M X	80	86	
Tumor Stage	Stage Ⅰ	140	140	168
	Stage Ⅱ	66	54	58
	Stage Ⅲ	38	42	0
	Stage Ⅳ	8	17	0

### 2.2 Univariate Cox proportional hazards regression

To identify genes closely related to OS from the train dataset, we performed univariate Cox proportional hazard regression study as previously described in Jin‐Cheng et al.^16^ *p* < 0.05 was the threshold.

### 2.3 Data analysis on copy number variation

GISTIC detects both focal and broad (probably overlapping) reappearing events. We used GISTIC 2.0^17^ software to determine genes with significant amplification or deletion. The parameter thresholds for fragments with amplification or deletion lengths were ˃ 0.1 and *p* < 0.05.

### 2.4 Analysis of gene mutation

Significantly mutated genes in the MAF file of TCGA mutation data were screened by Mutsig 2.0 software. The threshold was set to *p* < 0.05.

### 2.5 Development of a prognostic immune gene signature

Genes significantly related to patient OS, amplification, deletions, and mutations were selected, and those showing prognostic significance were obtained applying randomized survival forest algorithm.^18^ According to Jin et al,^19^ random survival forest in R package was used for filtering genes, iterations number of Monte Carlo was 100, and the number of previous progressions was 5. Characteristic genes were defined as genes exhibiting relative significance higher than 0.4. Further Cox regression study based on multiple variables was performed, and the following risk scoring mode was built:

$$RiskScore=\sum_{k=1}^{N}Exp_{k}*e_{k}^{\mathrm{HR}}$$

In which N denotes quantity of prognosis‐related genes, $Exp_{k}$ refers to the expression level of prognosis‐related genes, and $e_{k}^{\mathrm{HR}}$ represents the assessed gene regression coefficient in the multivariate Cox regression study.

### 2.6 Analysis of functional enrichment

Pathway enrichment analysis of Kyoto Encyclopedia of Genes and Genomes (KEGG) and Gene Ontology (GO) was performed in the R package clusterprofiler,^20^ identifies over‐represented GO terms in biology‐related courses, cellular element and molecular function, and KEGG pathway. Here, in the study, FDR <0.05 represented statistics‐related significance.

GSEA^21^ was conducted using the JAVA program (http://software.broadinstitute.org/gsea/downloads.jsp) with the MSigDB^22^ on C2 canonical pathway gene set collection containing 1320 gene sets. After conducting 1000 permutations, gene sets of a *p* < 0.05 showing noticeable up‐regulation were filtered.

### 2.7 Statistical analysis

For comparing survival risk between the two risk groups, the Kaplan‐Meier (KM) curve was drawn using the score of median risk as a cutoff. For testing whether genetic markers were independent prognostic factor, a multivariate Cox regression study was performed. Statistical significance was determined when *p* < 0.05. AUC study was carried out in the R package pROC. The heat map was drawn using R package Pheatmap. Default parameter was used in all analyses in R software version 3.4.3, unless otherwise specified.

## 3 RESULTS

### 3.1 Identification of gene sets in correlation with survival of LUAD cases

For samples of the TCGA train set, the study performed univariate regression to examine the relation between gene expression and patients’ overall survival (OS). Among the 2071 prognostic genes identified, 1161 genes were of HR<1, while 910 genes were of HR>1. The top 20 genes were listed in Table 2.

**TABLE 2:** The top20 most relevant genes for OS

gene	HR	coefficient	Z‐score	*p* value
ENSG00000107859	1.48694912	0.39672645	5.72186084	1.05E−08
ENSG00000148704	1.434454599	0.360784707	5.57425514	2.49E−08
ENSG00000107984	1.533873881	0.427796484	4.962716871	6.95E−07
ENSG00000138829	1.422130151	0.352155854	4.647037727	3.37E−06
ENSG00000156687	1.46213808	0.379899803	4.635544845	3.56E−06
ENSG00000140478	1.425955115	0.354841845	4.561167459	5.09E−06
ENSG00000178462	1.400199512	0.336614735	4.294593782	1.75E−05
ENSG00000185888	1.376019257	0.319194734	4.263138276	2.02E−05
ENSG00000163975	1.531203385	0.426053952	4.23019025	2.33E−05
ENSG00000159217	1.353621993	0.302783958	4.06409894	4.82E−05
ENSG00000165891	1.456027917	0.375712123	4.011611337	6.03E−05
ENSG00000133466	1.506641141	0.409882763	4.007282786	6.14E−05
ENSG00000266265	1.354880774	0.303713461	3.976929914	6.98E−05
ENSG00000106031	1.355842036	0.30442269	3.962817439	7.41E−05
ENSG00000161714	1.454645204	0.374762025	3.892559471	9.92E−05
ENSG00000251258	1.325733251	0.281965703	3.885360438	0.000102178
ENSG00000121691	0.659952564	−0.415587319	−3.877219552	0.000105657
ENSG00000179241	1.462860113	0.380393501	3.8721129	0.000107896
ENSG00000145192	1.33684358	0.290311298	3.865936536	0.000110664
ENSG00000197213	1.315374686	0.274121558	3.816051598	0.000135604

### 3.2 Genomic variation identification with gene set

**FIGURE 2:**
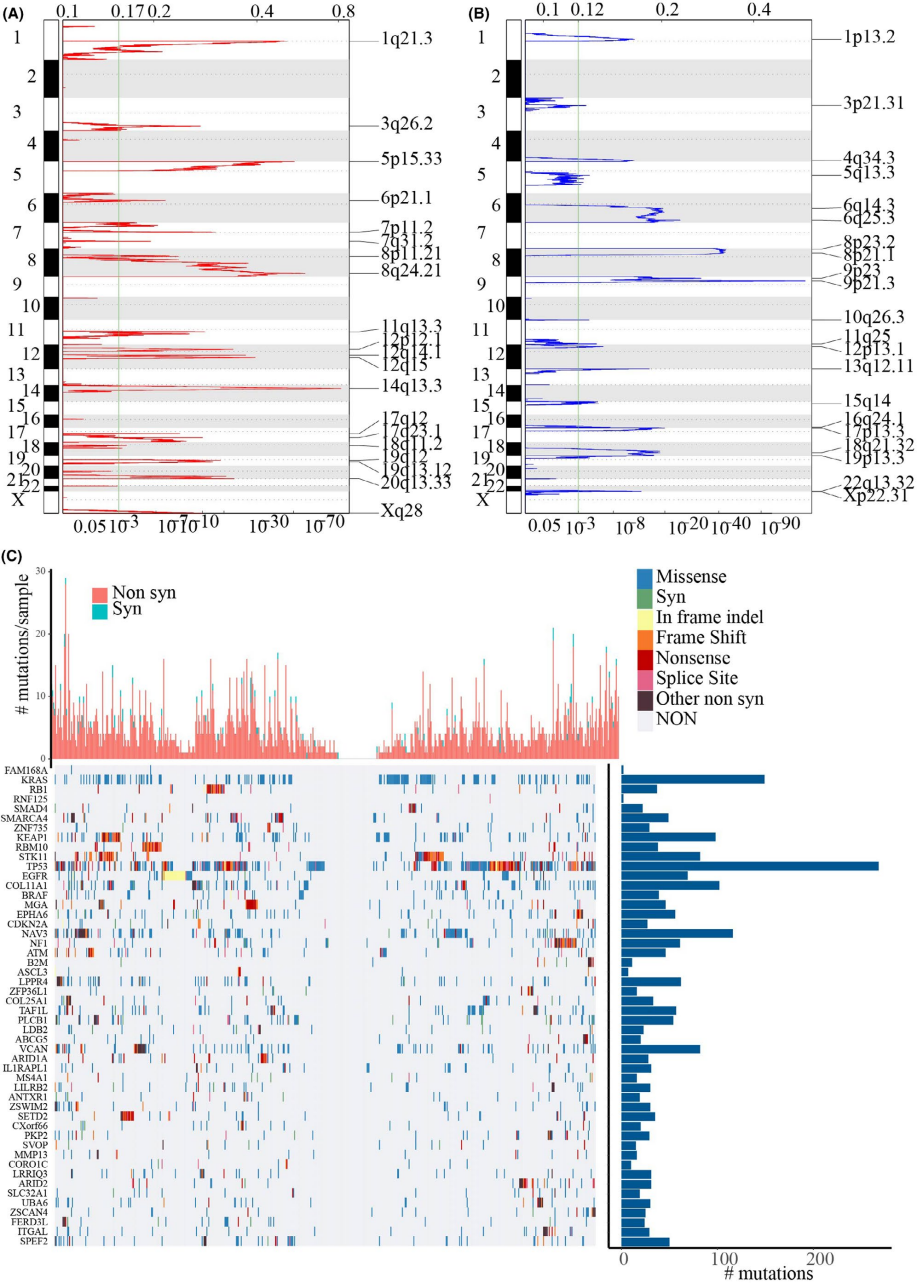
mRNAs located in the focal CNA peaks are LUAD‐related. Scores and false‐discovery rates (q values) from GISTIC 2.0 for alterations (x‐axis) plotted against genome positions (y‐axis); dotted lines indicate the centromeres. A: The amplifications (red) of genes. B: The deletions (blue) of genes. The green line represents 0.25 q value cutoff point that determines significance. C: The most significant mutation in the top 50 genes, the upper histogram shows the total number of nonsynonymous and synonymous mutations in each of these 50 genes, the histogram on the right shows the number of samples in which 50 genes have mutated in all samples. Different colors in the heat map indicate the type of mutation, and the gray color means no mutation

Genes showing significant deletion or amplification were identified by GISTIC 2.0 using copy number variation data from TCGA. There were 20 significantly amplified fragments in the genome of 230 genes (Figure 2A); noticeably, these fragments involved many important genes such as significant amplification of KRAS in the 12p12.1 segment (q value = 2.42E‐12), significant amplification of EGFR in the 7p11.2 segment (q value = 2.16E‐09), and significant expansion of ERBB2 in the 17q12 segment (q value = 2.01E‐05). Moreover, there were 21 significant deletion fragments (Figure 2B) involving 1878 genes, and among these genes, CD1 showed significant absence in the 1p13.2 segment (q value = 2.63E‐05), APC had significant deletion in the5q13 segment (q value = 0.017957), and CDKN2B also showed a great deletion in the 9p21.3 segment (q value = 1.08E‐81). Mutsig2 was used to screen significantly mutated genes using TCGA mutational annotation data; here, a total of 438 genes with significant mutation frequencies were detected. Figure 2C lists the top 50 genes in the sample showing the most significant framework insertions or deletions, missense mutations, synonymous mutations, nonsense mutations, framework shifts, other nonsynonymous, or cleavage sites. Among the 50 genes, KRAS, RB1, SMAD4, TP53, EGFR, and BRAF were closely involved in the LUAD development.

### 3.3 Functional analysis on genomic variant genes

**FIGURE 3:**
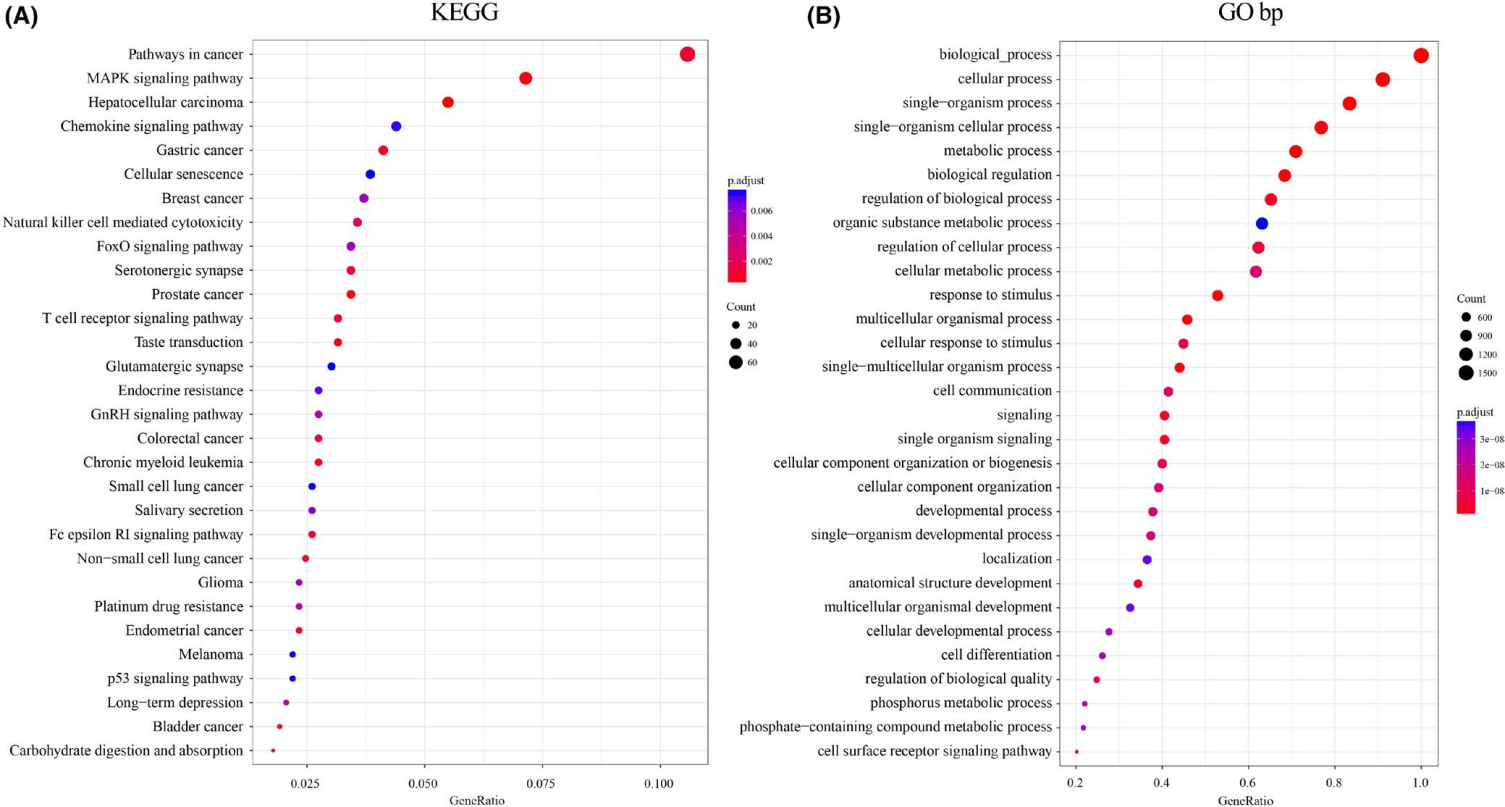
Functional enrichment analysis on 2261 genomic variant genes. A: Enriched KEGG biological pathways. B: Enriched GO terms in the “biological process” category. Different colors indicate different saliency, and different sizes reflect the number of genes

For investigating the functions of genes with genomic variations, 2261 deleted or amplified genes and significant mutant genes screened based on copy number variation were integrated. GO biological process and KEGG functional up‐regulation study was conducted on the 2261 genes. KEGG enrichment analysis revealed that natural killer cell‐mediated cytotoxicity, t‐cell receptor signaling pathway, MAPK signaling pathway, chemokine signaling pathway, Foxo signaling pathway, other KEGG biological pathways, and non‐small‐cell lung cancer were related to the cancer development (Figure 3A). In the category of biological process, the pathways were mainly enriched in metabolic process, cell communication, cell differentiation, developmental process, and other GO terms (Figure 3B). Interestingly, these above terms were closely correlated with cancer progress. Our data indicated that the genes of these genomic variants were linked to tumor development.

### 3.4 A 7‐gene signature for LUAD survival was built

**FIGURE 4:**
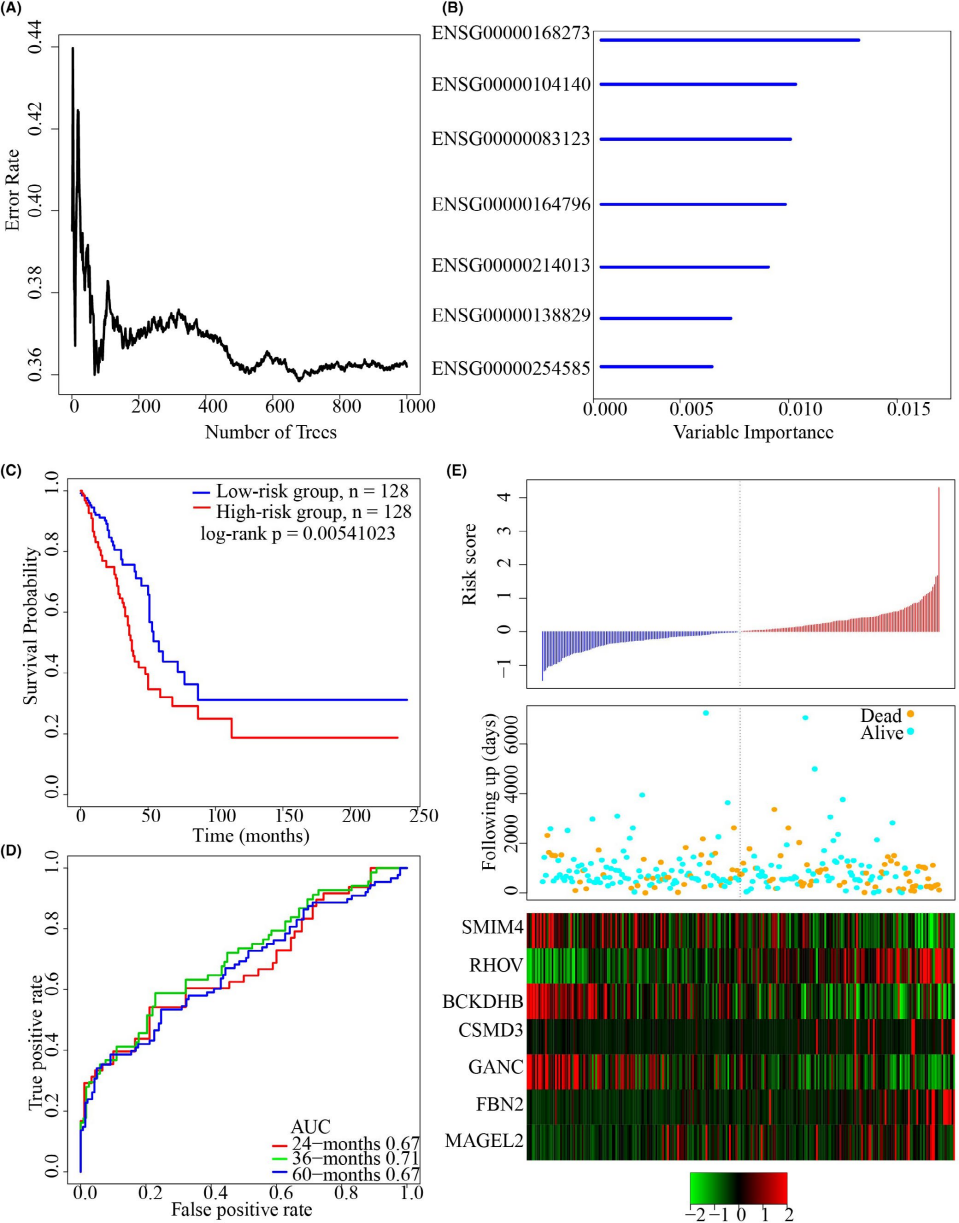
Establishment of a 7‐gene signature for LUAD survival. A: The relationship between the number of classification trees and the error rate. B: The order of importance of 7 genes out‐of‐bag. C: KM survival curve distribution of the 7‐gene signature in the TCGA training set. D: The ROC curve and AUC of the 7‐gene signature classification. E: The risk score, survival status and survival time, and the expressions of the 7 genes of the TCGA training set

Genomic variant genes and prognosis‐related genes were integrated, and a sum of 218 genes in the intersection of the three groups was selected as a candidate gene. Based on the relation of the quantity of classification trees and error rate, random forest was used in feature selection (Figure 4A). Here, genes with a significance greater than 0.4 were recruited to build a gene signature, and here, 7 genes were acquired (Table 3). The 7 genes were ranked for their out‐of‐bag value (Figure 4B). The 7‐gene signature was built with Cox regression analysis based on multiple variables as follow:

$$Risk_{7}=‐0.1242466*SMIM4+0.2498433*RHOV‐0.1681485*BCKDHB+0.2310252*CSMD3‐0.1523721*GANC+0.2032291*FBN2‐0.01186835*MAGEL2$$

**TABLE 3:** 7‐genes was significantly associated with the overall survival in the training‐set patients

Ensembl Gene ID	Symbol	HR	Z‐score	*p* value	Importance	Relative Importance
ENSG00000168273	SMIM4	0.78	−2.208803	2.72E−02	0.0119	1
ENSG00000104140	RHOV	1.30	2.641546	8.25E−03	0.009	0.755
ENSG00000083123	BCKDHB	0.78	−2.290767	2.20E−02	0.0087	0.7351
ENSG00000164796	CSMD3	1.33	3.658676	2.54E−04	0.0085	0.7152
ENSG00000214013	GANC	0.73	−2.486935	1.29E−02	0.0077	0.649
ENSG00000138829	FBN2	1.42	4.647038	3.37E−06	0.0056	0.4702
ENSG00000254585	MAGEL2	1.20	2.275242	2.29E−02	0.0051	0.4305

The sample risk score was obtained, and the samples were divided according to the medium the risk score (cutoff = −0.04131651). Patients’ prognosis in the two risk groups was significantly different (Figure 4C). Our result showed that the 3‐year AUC of the model was 0.71 in the training set (Figure 4D). Analysis on expression correlation of the 7 genes and risk score showed that high expression and high‐risk correlation of RHOV, CSMD3, FBN2, and MAGEL2 were risk factors, whereas low‐risk correlation of SMIM4, BCKDHB, GANC, and high expression was protective factors (Figure 4E).

### 3.5 The robustness of the 7‐gene signature was verified

**FIGURE 5:**
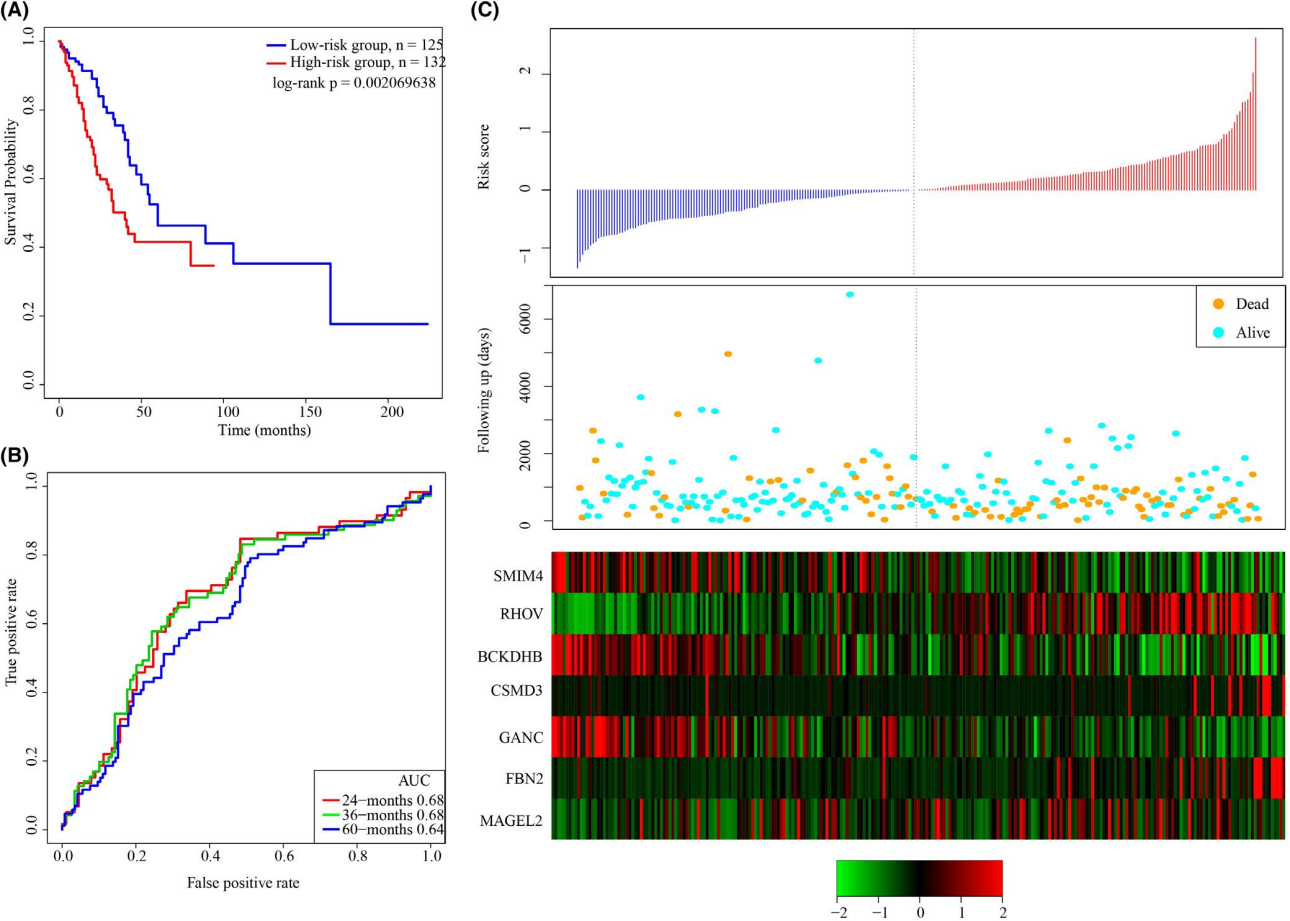
Robustness of the 7‐gene signal model was verified in test dataset. A: KM curve in the test set sample. B: The ROC curve and AUC of the 7‐gene signature classified in the test dataset. C: The relation between risk scores and the expressions of the 7 genes in the test set samples

To verify the robustness of the 7‐gene signature, the sample risk score in the test set was calculated. According to the threshold of the training set, we divided the samples into two groups, and noticeable prognostic diversifications between the 2 groups were observed (Figure 5A). Analysis of ROC showed a 5‐year AUC of 0.68 (Figure 5B). The risk score and the expression of the 7 genes were consistent in the training set (Figure 5C). Thus, the model was validated as an effective prognostic classifier in the TCGA dataset.

**FIGURE 6:**
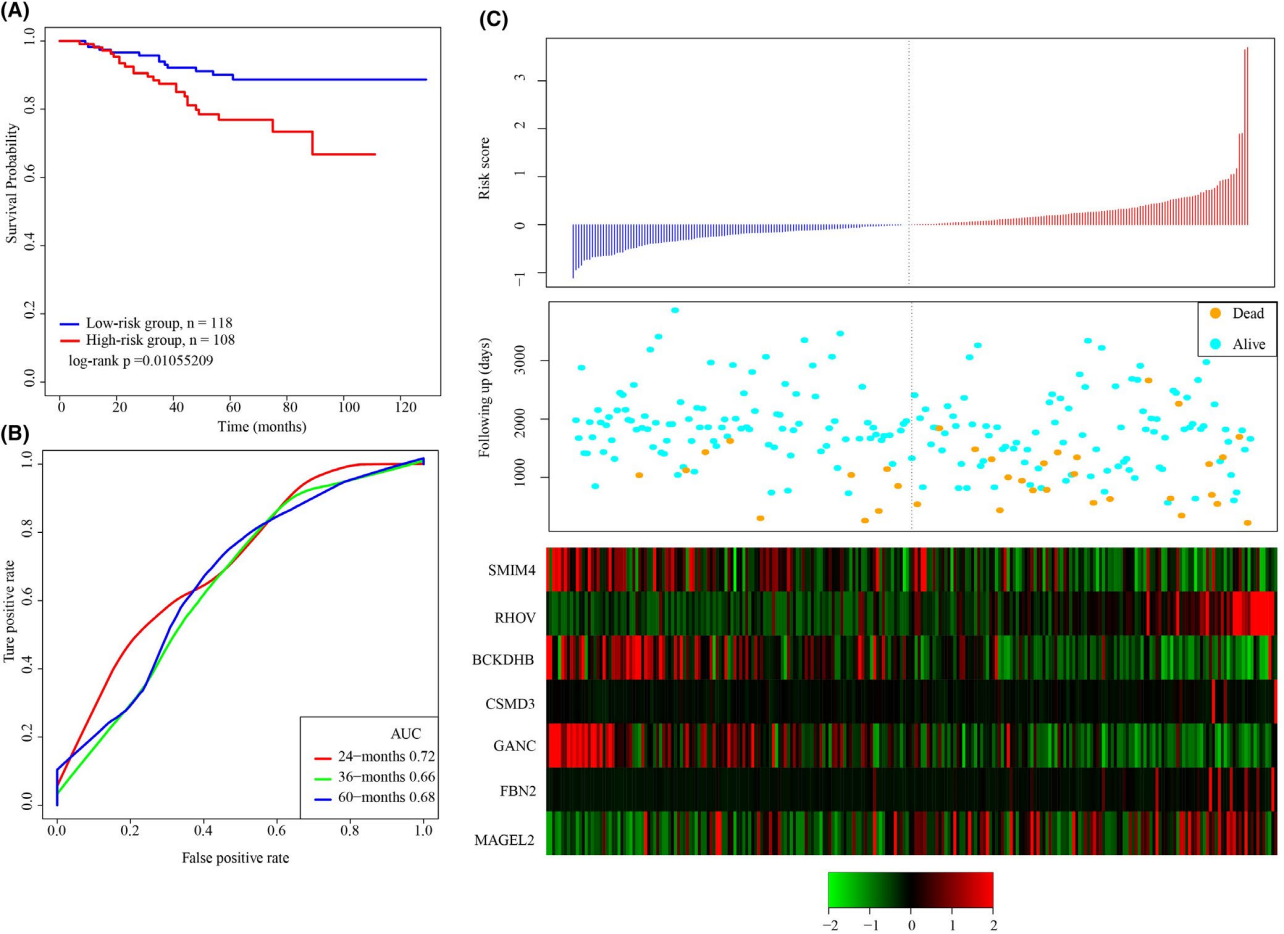
Robustness of the 7‐gene signal model was verified in GSE31210 dataset. A: KM survival curve distribution of the 7‐gene signature in GSE31210. B: ROC curves and AUC of the 7‐gene signature classification. C: The relationship of expressions of the 7 genes, survival time, risk score, and survival status in GSE31210

GEO platform was an external dataset for verifying the classification of the gene prediction system in different platforms. The signature was applied to determine sample risk score. The cutoff of the training set was the threshold for grouping samples into low‐ and high‐risk groups. We found that patients’ prognosis was evidently better in the low‐risk group than the high‐risk group (Figure 6A). Moreover, ROC analysis showed a 3‐year AUC of 0.66, which was similar to the training set (Figure 6B), and the relationship between the risk score and the expressions of the 7 genes was also consistent in the training set (Figure 6C). To conclude, our 7‐gene signature model showed a prognostic significance in both external and internal datasets.

### 3.6 The 7‐gene signature model was clinically independent

To identify whether the 7‐gene signature system was independent in clinical applications, the 95% confident interval (CI) of HR, relevant hazard ratio (HR), and p value were determined by performing multivariate and univariate Cox regression in the TCGA test set, TCGA training set, and GSE31210 data with clinical information. We then analyzed the clinical information of TCGA, GSE65858 patients’ data covering tumor stage N stage, M stage, T stage, gender, age, and group information using our 7‐gene signature (Table 4). Analysis from univariate Cox regression showed that in the TCGA training set, high‐risk group, pathologic T3, pathologic N1, pathologic T2, pathologic N2/N3, tumor stage II, tumor stage III, tumor stage IV displayed noticeable associations with survival. However, from multivariate Cox regression analysis, only high‐risk group (*p* = 3.40E‐05, CI = 1.52–3.27, HR = 2.24, 95%) was clinically independent. In the TCGA test set, from the results of univariate Cox regression analysis, we observed that pathologic N1, pathologic T4, pathologic T3, pathologic N2/N3, tumor stage II, tumor stage IV, tumor stage III, and high‐risk group were evidently related to survival. Multivariate Cox regression demonstrated that pathologic T3, pathologic N1, and high‐risk group (*p* = 0.002, 95% CI = 1.25–2.79, HR = 1.86) were clinically independent. In GSE65858, from the data of univariate Cox regression analysis, it could be found that tumor stage II and high‐risk group were noticeably correlated with survival. Corresponding multivariate Cox regression study demonstrated that high‐risk group (*p* = 0.008, CI = 1.17–2.91, HR = 1.85, 95%) and tumor stage II were clinically independent. Hence, the 7‐gene prediction system was a prognostic marker independent of other clinical factors.

**TABLE 4:** Univariate and multivariate Cox regression analysis identifies clinical factors and clinical independence associated with prognosis

Variables	Univariate analysis			Multivariable analysis		
	HR	95%CI of HR	*p* value	HR	95%CI of HR	*p* value
TCGA training datasets						
7‐gene risk score						
Low‐risk group	1 (reference)			1 (reference)		
High‐risk group	2.72	2.01–3.68	9.530E−11	2.24	1.52–3.27	3.40E−05
Age	1.01	0.99–1.03	0.606	1.01	0.99–1.03	0.277
Female	1 (reference)			1 (reference)		
Male	0.84	0.56–1.26	0.40	0.85	0.54–1.31	0.468
Pathologic T 1	1 (reference)			1 (reference)		
Pathologic T 2	1.73	1.02–2.93	0.042	1.36	0.77–2.37	0.286
Pathologic T 3	2.99	1.39–6.38	4.86E−03	1.56	0.59–4.11	0.370
Pathologic T 4	1.78	0.52–6.08	3.56E−01	0.83	0.18–3.77	0.808
Pathologic N 0	1 (reference)			1 (reference)		
Pathologic N 1	2.34	1.48–3.70	2.60E−04	1.22	0.50–2.92	0.657
Pathologic N 2/ N 3	4.10	2.34–7.16	7.51E−07	1.51	0.32–6.91	0.598
Pathologic M 0	1 (reference)			1 (reference)		
Pathologic M 1/ M X	1.17	0.73–1.88	5.13E−01	1.14	0.66–1.96	0.64
Tumor stage Ⅰ	1 (reference)			1 (reference)		
Tumor stage Ⅱ	2.77	1.69–4.50	4.50E−05	1.87	0.73–4.77	0.192
Tumor stage Ⅲ	4.50	2.60–7.79	7.53E−08	2.57	0.49–13.32	0.26
Tumor stage Ⅳ	5.80	2.38–14.07	1.03E−04	2.92	0.75–11.33	0.12
Validation cohort, TCGA test datasets, GSE31210						
TCGA test datasets						
7‐gene risk score						
Low‐risk group	1 (reference)			1 (reference)		
High‐risk group	1.88	1.31–2.71	6.65E−04	1.86	1.25–2.79	0.002
Age	1.01	0.98–1.03	0.565	1.01	0.98–1.03	0.334
Female	1 (reference)			1 (reference)		
Male	1.36	0.89–2.06	0.147	1.21	0.77–1.88	0.397
Pathologic T 1	1 (reference)			1 (reference)		
Pathologic T 2	1.26	0.77–2.06	0.350	1.21	0.70–2.08	0.482
Pathologic T 3	3.07	1.49–6.30	0.002	3.89	1.53–9.86	0.004
Pathologic T 4	3.90	1.75–8.68	8.57E−04	1.55	0.59–4.02	0.368
Pathologic N 0	1 (reference)			1 (reference)		
Pathologic N 1	2.55	1.49–4.33	5.71E−04	2.72	1.07–6.88	3.49E−02
Pathologic N 2/ N 3	2.39	1.40–4.04	1.23E−03	1.70	0.50–5.73	3.95E−01
Pathologic M 0	1 (reference)			1 (reference)		
Pathologic M 1/ M X	0.95	0.61–1.47	0.81	0.68	0.40–1.16	0.163
Tumor stage Ⅰ	1 (reference)			1 (reference)		
Tumor stage Ⅱ	2.15	1.23–3.74	0.007	0.58	0.22–1.52	0.270
Tumor stage Ⅲ	2.93	1.71–5.00	8.23E−05	0.96	0.24–3.69	0.950
Tumor stage Ⅳ	3.04	1.50–6.16	2.02E−03	2.04	0.68–6.10	0.203
GSE31210						
7‐gene risk score						
Low‐risk group	1 (reference)			1 (reference)		
High‐risk group	1.94	1.31–2.86	8.80E−04	1.85	1.17–2.91	0.008
Age	1.03	0.98–1.08	3.06E−01	1.04	0.98–1.08	0.167
Female	1 (reference)			1 (reference)		
Male	1.52	0.78–2.96	2.19E−01	1.23	0.62–2.44	0.552
Tumor stage Ⅰ	1 (reference)			1 (reference)		
Tumor stage Ⅱ	4.23	2.18–8.24	*p* ≤ 0.001	3.85	1.96–7.55	8.67E−05

### 3.7 Validation of 7‐gene signature model in CNV and mutation datasets

**FIGURE 7:**
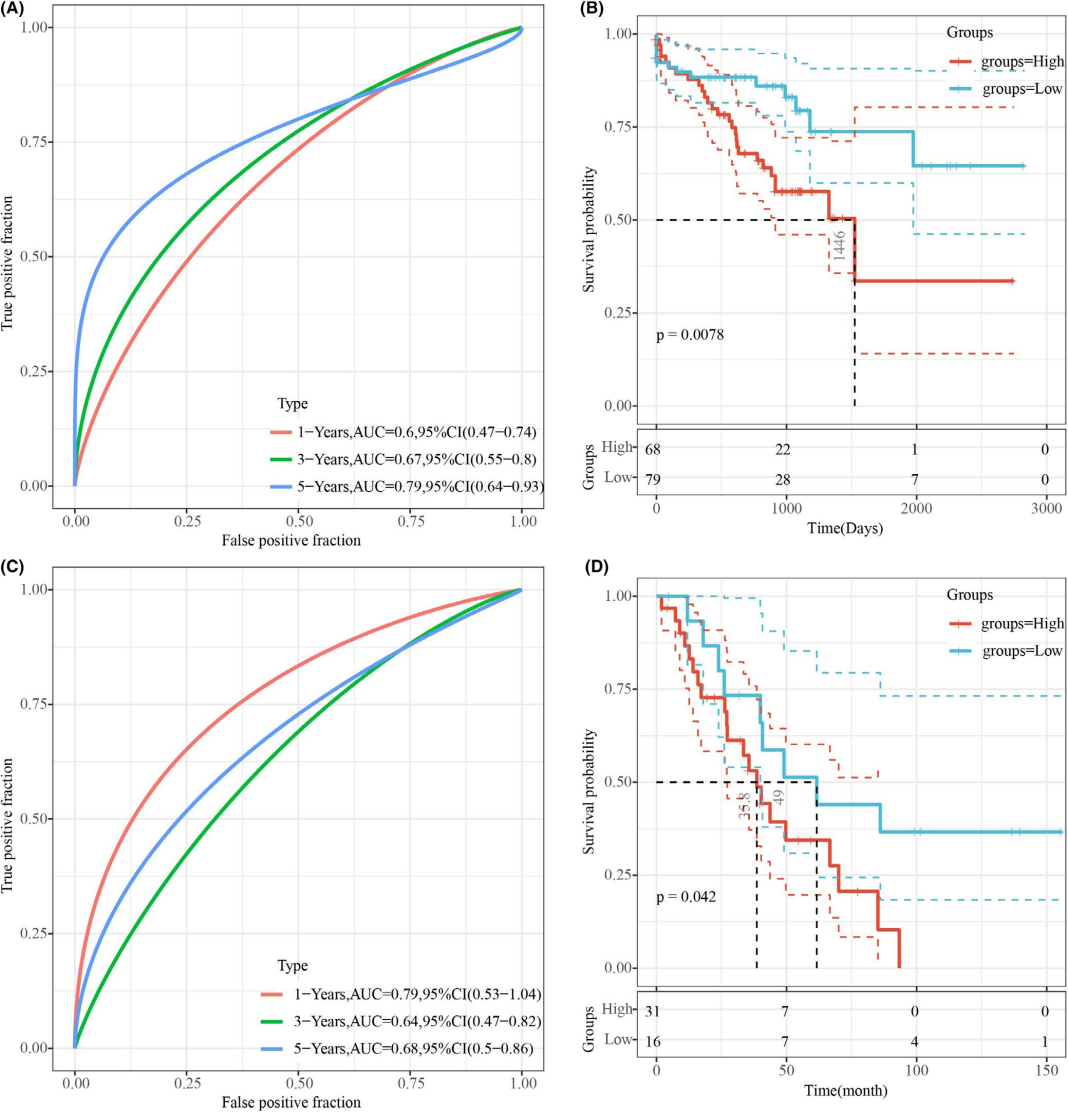
7‐gene signature was validated in mutation dataset and copy number variation dataset. A: ROC analysis of mutation characteristics of LUSC‐KR cohort; B: K‐M curve of mutation characteristics of LUSC‐KR cohort; C: ROC analysis of copy number characteristics of GSE36363 queue; B: K‐M curve of copy number characteristics of GSE36363 queue

We added additional validation of CNV and mutation datasets. Specifically, we derived the lung adenocarcinoma samples from ICGC mutation dataset LUSC‐KR (https://dcc.icgc.org/projects/LUSC‐KR), to verify that these gene mutations are in the relationship with the prognosis. Multivariate regression analysis was used to calculate the mutation characteristics score of 7 genes in each patient. ROC analysis showed that the 5‐year AUC was 0.79, and the 1‐year AUC was 0.6 (Figure 7A), suggesting that the mutation characteristics of these 7 genes could effectively evaluate the prognosis of patients. The R software package maxstat was used to classify patients, and the prognosis of patients with high scores was significantly worse than that of patients with low scores (Figure 7B).

We also obtained from the GEO database of lung adenocarcinoma of CNV data queue GSE36363 (https://www.ncbi.nlm.nih.gov/geo/query/acc.cgi?acc=GSE36363), extracted copy number of seven genes. The copy number characteristic scores of seven genes were calculated for each patient using the same method. ROC analysis showed that the 5‐year AUC was 0.68 and 1‐year AUC was 0.77 (Figure 7C), which suggested that the prognosis of patients could be effectively evaluated based on the copy number characteristics of these 7 genes. Patients were classified by maxstat, R software package. Patients with high scores had significantly worse outcomes than those with low scores (Figure 7D).

### 3.8 Enriched pathway differences in the two risk groups analyzed by GSEA

**FIGURE 8:**
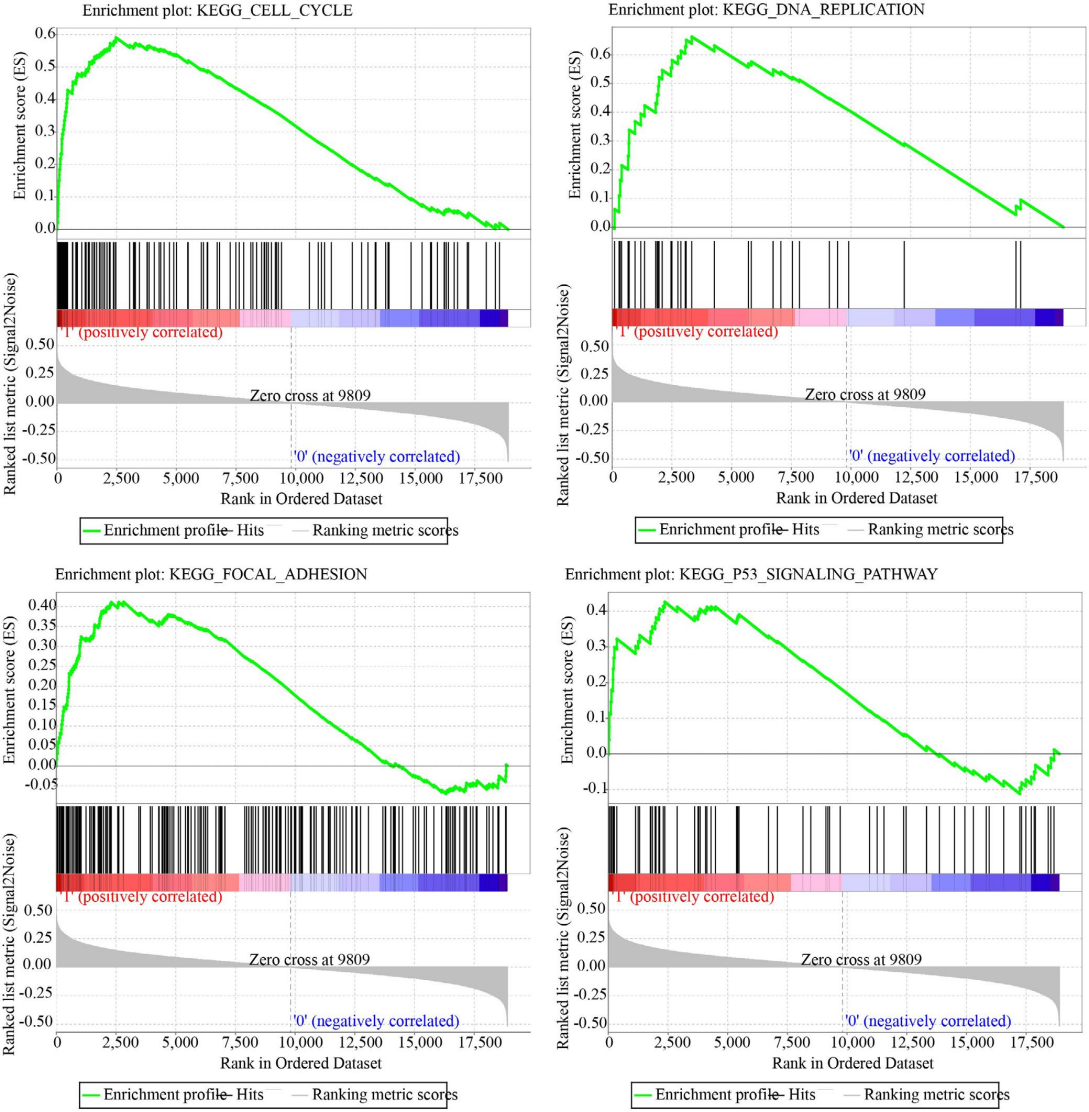
GSEA enrichment results of four pathways, cell cycle, DNA replication, focal adhesion, and P53 signaling pathway

GSEA in the TCGA training set was used to analyze significantly enriched pathways in the two groups (low‐risk and high‐risk groups), and we obtained a total of 30 significantly enriched pathways, including pathways (eg, P53 signaling pathway, DNA replication, focal adhesion, cell cycle), which were tightly linked to the progress and metastasis of LUAD. The above pathways were greatly enriched to the samples of the high‐risk group (Figure 8).

## 4 DISCUSSION

LUAD is a highly heterogeneous cancer, as LUAD patients with similar TNM staging often develop different survival conditions. Traditional clinical pathology indexes (eg, vascular invasion, tumor size, TNM staging, and portal vein tumor thrombus) are hard to fit the present need for an accurate prediction of individual treatment outcomes because currently there is no effective one‐size‐fits‐all treatment.^23^, ^24^ Prognostic molecular markers can show the biology features of tumors and are of great significance for individualized treatment for LUAD and its prevention. This study analyzed the expression profiles of 739 LUAD samples collected from TCGA and GEO, and developed a robust 7‐gene signature, which was independent of clinic‐related elements but was predictive of the OS of LUAD patients. Gene signature models are now applied in clinical practice, for example, Oncotype DX expressed by 21 genes is indicative of disease recurrence score,^25^, ^26^, ^27^ and Coloprint with an 18‐gene signature could predict colon cancer.^28^, ^29^, ^30^ These findings demonstrated that profiling of gene expression to discover markers for cancer prognosis has become an effective method in high‐throughput molecular identification. In the study of Wang et al,^11^ weighted gene correlation network study was employed to construct a 4‐gene model for evaluating overall survival condition of patients with LUAD and lymph node metastasis; interestingly, the model AUC reached around 0.7 and was verified in an external dataset. However, their verification dataset (*N* = 140) and training dataset (*N* = 156) were small, which may lead to bias in their results. In the current study, the AUC was close to 0.7 in test set (*N* = 257), the training set (*N* = 256), and validation set (*N* = 226) using our 7‐gene signature prediction. The 7‐gene signature showed a high AUC but involved fewer genes; moreover, as these genes had genomic mutation abnormalities that were easy to be clinically detected, our 7‐gene signature had a high potential in clinical transformation.

In our 7‐gene signature, RHOV, CSMD3, FBN2, and MAGEL2 were risk factors, and SMIM4, BCKDHB, and GANC were protective factors. It has been reported that RHOV is overexpressed in non‐small‐cell lung cancer.^31^ CSMD3 mutations are related to favorable clinical outcomes in esophageal cancer.^32^ Mutation of CSMD3 in non‐small‐cell lung cancer leads to increased proliferation of airway epithelial cells,^33^ and abnormal methylation of FBN2 is a biomarker for lung cancer.^34^, ^35^ Our research provides a better understanding for subsequent study on the clinical significance and biological role of the 7 genes. MAGEL2, SMIM4, BCKDHB, and GANC have not been previously found to be related to tumors, and they were the first confirmed as novel prognostic markers for LUDA in this study. Furthermore, the current GSEA analysis also showed that pathways enriched by the 7 genes were closely associated with biological processes and pathways in LUDA progress. According to the current findings, the 7‐gene signature could be clinically applied, providing possible targets for diagnosis of LUDA patients.

Though this study identified possible genes for tumor prognosis in large samples through bioinformatics, several limitations should be noted. For instance, our samples had insufficient clinical follow‐up data. Thus, some elements, for example, other health conditions of patients in distinguishing prognostic biomarkers, were not taken into account. Also, the outcomes achieved only with bioinformatics may be inadequate, which requires experimental confirmation with a larger sample size.

To sum up, we developed a 7‐gene signature stratification system with a high AUC in the validating sets and the training sets, and showed independence of clinical features. In comparison with other clinical features, the 7‐gene classifier can improve the prediction of survival risk. Hence, the classifier developed in this research can serve as a molecular diagnostic marker for evaluating the prognostic risk of cases with LUDA.

## CONFLICT OF INTEREST

The authors declare no conflicts of interest.

## AUTHOR CONTRIBUTIONS

Huaxing Huang and Surong Zhang involved in conception and design of the research. Xueni Zeng and Shaona Lin acquired the data. Shaona Lin involved in the statistical analysis. Minchao Liang involved in analysis and interpretation of data. Surong Zhang drafted the article. Huaxing Huang revised the article for important intellectual content. All authors read and approved the final article.

## Data Availability

The analyzed data generated during the study are available from the corresponding author on reasonable request.
